# Neuro-Fuzzy Modelling of the Metallic Surface Characterization on Linear Dry Contact between Plastic Material Reinforced with SGF and Alloyed Steel

**DOI:** 10.3390/ma11071181

**Published:** 2018-07-10

**Authors:** Victor Vlădăreanu, Lucian Căpitanu, Luige Vlădăreanu

**Affiliations:** Institute of Solid Mechanics of the Romanian Academy, C-tin Mille 15, 010141 Bucharest, Romania; victor.vladareanu@vipro.edu.ro or vladareanuv@gmail.com (V.V.); lucian.capitanu@yahoo.com (L.C.); luigiv2007@gmail.com (L.V.)

**Keywords:** ANFIS, fuzzy logic, clustering, neural networks, robotics and contact wear

## Abstract

This paper presents the modelling of wear data resulting from linear dry contact using artificial neural networks (ANN) and adaptive neuro-fuzzy inference systems (ANFIS) with the aim of constructing predictor models for the depth and volume of the wear scar, with great impact in the characterization of new industrial processes utilizing existing materials. The dataset is the result of laboratory testing, presenting both numerical and categorical variables whose inclusion into the model allows for a number of possibilities. The width of the wear scar was measured on a microscope, and its depth was calculated. A multitude of experimental tests was performed with normal loads and different speeds, which led to some conclusive results, but in some cases, with relatively high variance. Various options for the automatic generation of fuzzy inference systems were also approached (genfis2). The innovative approach was compared with a baseline model featuring multivariate linear regression optimized using gradient descent, drawing on previous experimentation on the same dataset. The models developed can be implemented in future research and in practical applications under similar conditions, aiming to optimize performance by applying Computer Science. The obtained results lead to highly accurate prediction models which are further integrated into various metallic surface characterizations in the wear process for tribological and robotics research in new industrial processes using short glass fiber reinforced polymers.

## 1. Introduction

The paper presents new intelligent analytical methods for the characterization of wear in thermoplastic materials armed with short glass fibers (SGF) and steel in a dry contact wear scenario, applied to new industrial processes using existing materials.

Wear phenomena are very complex within injection or extrusion machine cylinders. The tribological hostile environment, high temperature, and corrosive chemical compounds increase this complexity. In the case of processing thermoplastic materials with short glass fiber fillings, complexity is further increased because of their significant abrasion.

The mechanical interaction in the form of wear, which always appears between two or multiple bodies when there are relative speeds, sliding, rolling, pivoting, and so on, defines friction wear. The process complexity is determined by the wear indicators, among which are the linear wear intensity, volumetric wear intensity, gravimetric wear intensity, wear coefficient, wear sensibility coefficient, and apparent energy density.

Previous studies on friction couples with linear contact on thermoplastic material armed with short glass fibers (SGF) and steel in a dry friction context have shown in experiments that even under normal, relatively small loads, large contact pressures and therefore very high contact temperatures may appear, which are close or may even exceed the transformation temperature of the plastic material.

These lead to the need to approach modelling as a dependency between the various variables of interest involved in the friction process and metallic surface characterization in the wear process using advanced statistical and optimization algorithms on a dataset obtained from hardware simulation. The subject draws from growing interest from the research community with the advent of highly advanced, intelligent classification; optimization and regression algorithms; and the wide impact of metallic surface characterization applications in the wear process, emphasizing the abrasive, adhesive, and corrosive wear.

Zhang et al. [1] analyzed the artificial neural network prediction of erosion wear of the polymer. Three independent sets of measurement data were used and the characteristic properties of erosive wear of these polymers to prepare and test the neural networks were explored. For the first two examples of materials, the angle of impact of solid particle erosion and some characteristic properties were selected as the input variables of the ANN.

Similar directions were investigated in Panda et al. and Flepp [2,3] regarding the potential of supervised or unsupervised learning and modelling the results of friction, while surveys by Ripa and Frangu [4] dealt with the various possibilities for undertaking this task. Finally, the paper builds upon previous work done by authors Rus et al. [5]. The importance of the subject matter is illustrated by its use in building artificial joints and prosthesis—Căpitanu et al., Al-Zubaidi et al. [6,7], among others. Căpitanu et al. [6] presented an analytical qualitative–quantitative correlation of friction and wear processes of steel surfaces in linear dry contact with SGF that reveals the nonlinearity of tribological processes in this case.

Wear processes, similar to fabrication processes, involve very complex and nonlinear phenomena. Consequently, analytical models are difficult or impossible to come by. However, improvements in the performance and reliability of mechanical equipment and production instruments require precise modelling and prediction of the wear phenomenon. Artificial neural networks (ANNs) and the related methods investigated in this paper, such as neuro-fuzzy inference systems, possess many desirable properties for modelling systems and processes: the ability to approximate universal functions, learning from experimental data, high tolerance for lacking or noisy data, and good capacity for generalization.

Artificial neural networks are the driving force behind the current advances in artificial intelligence, with useful applications in virtually every computational field. They rely on successively improving a network of weights attached to hidden units called neurons. Neural networks work by solving for the best dynamic weights of a hidden layer of neurons, which determine the strength with which these are fired [8]. While solving for a linear or polynomial regression model provides an explicit relationship between dependent and independent variables, it may be that an implicit representation model such as a neural network would yield better results.

Shukla [9] reported an overview of the applications of artificial neural networks in the processing domain. The property of learning and nonlinear behavior makes them useful for complex nonlinear process modelling, better than analytical methods. They are useful in some specific points in the field: Processes and wear particles, manufacturing, friction parameters, and defects in mechanical structures. Rao et al. [10] worked with rolling element bearings, which are widely used in almost all global industries. Their proactive strategy was to minimize the imminent failures in real time and at minimal cost. Innovative developments have been recorded in the technology of artificial neural networks (ANN). Chen and Savage [11] described an approach to fuzzy networks for a recognition system of multilevel surface roughness (FN-M-ISRR), whose aim was to predict the surface roughness (Ra) under multiple cutting condition, determined by the material of the tool, the tool size, etc.

The same nonlinearity was found by Căpitanu et al. [12] in the behavior of the UHMWPE tibia insert of the total knee prostheses, and Vlădăreanu et al. [13] applied ANN to a versatile intelligent portable robot control platform based on cyber physical systems principles.

The baseline model considered for expressing a dependency between the various variables of interest involved in the friction process and metallic surface characterization in the wear process is a multivariate linear regression. The first step in a multiple variable regression model is to normalize the features and then run a batch gradient descent algorithm on the data, where each iteration minimizes the cost function by simultaneously updating all variable coefficients [14]. The linear regression model also includes regularization factors to prevent over-fitting. The regularization component is included in the cost function and provides a penalty for the data being fitted too closely using polynomial variables.

The resulting dataset is then used to train a linear regression model for each of the two considered dependent variables: Wear depth and wear volume. This model assumes the dependent variables to be a linear combination of the considered independent variables, speed and pressure, and an intercept term, which does not vary with the independent variables. The intercept term is added only for the linear regression problem since the neural network algorithm will do the same on its own.

The optimization problem is then to find the best coefficients that minimize the cost function, which gives a measurement of the difference between the empirical values of the two dependent variables and their estimates obtained through linear regression. Gradient descent is the algorithm used to iteratively arrive at the best possible set of variable coefficients. The learning rate for this version of gradient descent is set to 1.

For each dependent variable, the prediction is a dot-product of the independent variable values and its respective coefficient vector. The linear regression coefficients show the relative influence a certain independent variable has on the prediction of a dependent variable. The intercept term is a baseline starting point for the prediction models, being an aggregation of all the other factors not considered and the inherent randomness of the model.

Fuzzy logic and fuzzy inference systems extend regular logic systems by assigning a degree of membership to elements within sets, which proves to be a very useful ability for modelling complex, unknown, or dynamic systems. Fuzzy Logic has long been used in academia and in industry and is one of the more palpable staples of artificial intelligence in the world today. Fuzzy logic controllers have proven to be robust and relatively easy to design [15]. They seem to suffer from no one major flaw while providing a number of important benefits such as expert knowledge emulation. There are various algorithms for the optimization of fuzzy inference systems’ parameters such as genetic algorithms and neural networks. Neuro-fuzzy modelling (ANFIS) attempts to model the behavior of a given system for which arrays of input and output values are provided by creating a fuzzy inference system to produce similar results. The fuzzy inference system is then learned (i.e., its parameters are optimized) using an artificial neural network algorithm. This is a very convenient tool for simulating systems whose mathematical formulae are unknown or very complex. The overall concept is explained in further detail in [15,16], which are part of the authors’ previous work on the topic.

ANFIS implementations in the context of wear prediction deals mainly with fault prevention and monitoring. Zuperl and Cus [17] construct a tool monitoring system using a merged neural decision network and wear predictor for tool maintenance, while Lo [18] uses ANFIS and the grey system method for tool failure detection in single point turning operations. As relates to contact wear, Aliman et al. [19] investigate wear rate on a coated aluminum alloy. Shabani et al. [20] also obtained interesting results by combining ANFIS with particle swarm optimization in manufacturing wear resistant nano-composites.

The provenance of the initial fuzzy inference system is of great significance in designing an ANFIS algorithm. Fuzzy inference systems may be automatically obtained from the available data through a number of algorithms, which mainly attempt to group the available data-points into equivalent fuzzy sets and then deduce relations between them, which turn into a set of fuzzy rules. The resulting fuzzy inference systems (FIS) can be used to seed an ANFIS algorithm, that is, to have it start optimization from the previously obtained FIS. In fact, if no seed is specified, the ANFIS algorithm will itself use one of the methods, namely grid partitioning, to generate its starting FIS. As is traditional with ANFIS, all of the FISs investigated were of the Sugeno type due to complexity and computational constraints. In addition, because of the limited amount of data, the generated fuzzy inference systems themselves are considered as possible solutions for the proposed model, since ANFIS models are very susceptible to over-fitting the data in the present context.

The authors have had previous contributions to this and related topics in [5,6,11,12,15,16], among others. The original contribution of the current paper stems from implementing the proposed models on a recently obtained dataset, evaluating the different results and providing a comparison of the effect of the various algorithms. This entails a comprehensive comparative study on the same dataset, while varying the learning algorithm hyper-parameters and associated options, such as investigating the various methods used for fuzzy inference system generation. Finally, the successful models are to be chosen for future implementation in real world applications in the fields of Tribology and Robotics.

The remainder of this paper is divided as follows. Section 2 will discuss the experimental data, the type of considered variables, how the data is processed for the learning application, the procedures for the automatic generation of fuzzy inference systems from the available data, the neural networks, and the adaptive neuro fuzzy inference systems. Section 3 compares the results of the various model solutions on the test sets. Section 4 discusses these results and Section 5 draws conclusions on this and possible future work.

## 2. Materials and Methods

The study of injection and extruding processes for thermoplastic materials is a complex process due to the phenomena existing inside injection and extruding machines with a permanent interconnection of the influencing factors.

Starting from the material selection stage, either fine dust, or the quantity of short glass fibers (SGF) used, adding materials such as TiO_2_ (titanium dioxide) or graphite fibers, the technological process implies transforming the material from a solid to a plastic/liquid phase, which is achieved at temperatures above 1600 °C, with the material suffering deformation, pressing, and heating depending on the machine and the technological process.

In addition to the material complexity and the preparation for injection, an important part is played by determining a predictive model for the wear process in order to increase wear resistance of the work surfaces of the injection and extruding machines.

These are some of the considerations for the neuro-fuzzy modelling of wear data resulting from linear dry contact using artificial neural networks (ANN) and adaptive neuro-fuzzy inference systems (ANFIS).

**Figure 1:**
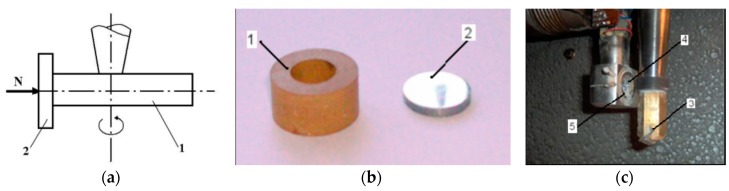
The functional diagram of the friction couple (**a**) the coupling elements (**b**) mounting the coupling to experimental equipment (**c**) movement of the bush against the disc, where 1—cylindrical thimble; 2—steel disc sample; 3—fastening nut; 4—carrier; 5—prism to ensure parallel contact.

Wear performance of the two steel alloys, C120 and Rp3, has been previously studied in the case of linear dry contact with each polymer (polyamide and polycarbonate) reinforced with different percentages of short glass fibers (SGF). The functional diagram of the friction couple is presented in Figure 1, from Căpitanu et al. (2014), where it looks at the linear contact. The friction couple comprises a cylindrical plastic liner and a flat polished steel hardened sample.

The wear scar occurs by penetration of the cylindrical liner, under the influence of the normal load, in the flat sample material. In theory, the holding thimble is considered as rigid and relatively small in view of the backside imprint, so it can be considered as made up of a sum of cylinder areas. This is illustrated schematically in Căpitanu et al. [11].

We considered the following three polymers:

- Nylonplast AVE Polyamide + 30% glass fibres; *E*_2A_ = 40.25 MPa.
- Noryl Polyamide + 20% glass fibres; *E*_2B_ = 31.76 MPa.
- Lexan Polycarbonate + 20% glass fibres; *E*_2C_ = 42.08 MPa.

Numerical values were determined by the elasticity modules (*E*) listed above, and the deformed liner radii (*r*_2_), imposing *p*_*max*_ is provided as $p_{max}<0.5\;H$, where *H* is the Brinell hardness for the plastic liner, enough so that it will not be crushed. The approximate depth of the wear scar is calculated with the relation: *h ≈ l*^2^*/*8*r*_2_.

The imposed condition allows the following values of the maximum contact pressure of the dry linear couplings contact to be established, in the case of three plastic materials (A, B, C) reinforced with SGF, the five normal loads (contact pressures), indexes 1 to 5 of notations of the pressures that have been subjected to tests, for each of the seven relative sliding speeds used (18.56; 27.85; 37.13; 46.41; 55.70, 111.4; and 153.57 cm/s):

*p*_A1_ = 16.3 MPa; *p*_A2_ = 23.5 MPa; *p*_A3_ = 28.2 MPa; *p*_A4_ = 32.6 MPa; *p*_A5_ = 36.4 MPa

*p*_B1_ = 12.3 MPa; *p*_B2_ = 17.4 MPa; *p*_B3_ = 21.4 MPa; *p*_B4_ = 24.6 MPa; *p*_B5_ = 27.6 MPa

*p*_C1_ = 16.9 MPa; *p*_C2_ = 23.9 MPa: *p*_C3_ = 29.3 MPa; *p*_C4_ = 33.8 MPa; *p*_C5_ = 37.8 MPa

After inspecting and measuring the wear scars of the metal surfaces, the widths of each wear scar were measured and their volume was calculated (the amount of material lost through wear). Their variation curves were also traced depending on the applied load (contact pressure), the relative speed of sliding contact with the temperature specification of the optical image and the presentation of the scar. This quantitative–qualitative assessment was presented in Căpitanu et al. [11]. All tests took place for 60 min, so that the calculated wear volumes are actually wear rates.

The increased friction coefficient entails increasing the wear rate and contact temperature, but after our data, it has not yet been possible to establish a mathematical relationship between the two sizes, which is widely recognized. This is why a suggestive graphical representation was sought to provide a qualitative correlation between the two sizes that relates them to the contact temperature and based on which to determine a quantitative correlation.

All the variation curves of the output parameter of the frictional system (amount of wear, depth of wear, friction coefficient, contact temperature) depending on input parameters, normal load (contact pressure), the sliding speed while maintaining the steady state surface (roughness *R*_a_), shows a strong nonlinearity due to the behavior of the elastic-plastic polymers tested. In this situation, we tried an approach to model metal surface wear through advanced data fitting algorithms, because of their ability to model very complex and strongly nonlinear phenomena. This was the qualitative–quantitative analytical approach previously achieved. The graphic processing of these results was presented in Căpitanu et al. [5].

The data for metallic surface characterization on linear dry contact between plastic material reinforced with SGF and alloyed steel was obtained through experiments run on friction couples with linear contact using three different types of polymers on two different types of steel variants. Aside from alternating the materials used, the speed and pressure applied to them were varied under the same operating conditions. This was done with regard to the particulars of each material combination and the levels of speed and pressure.

The method used approaches of the study of wear on a metallic surface in the case of dry linear contact, plastic reinforced with SGF on surfaces of alloyed steel, C120 and Rp3, through the method of artificial neural networks. This is necessary because the wear processes involve very complex and powerfully nonlinear phenomena, and analytic models are difficult or impossible to obtain. Furthermore, the multiple inputs (normal load, contact pressure, sliding speed, measured contact temperature, materials properties) and outputs (width and depth of the wear scar, contact temperature) influence each other continually.

The resulting dataset includes the following information, seen in Table 1.

For each of the variables considered, the table describes the following characteristics:

- Mode is whether the variable is treated as a dependent or an independent variable. The contention of any modelling technique is that the dependent variables can be represented as some relation, whether explicit or implicit, of the independent variables. In the present context, the paper investigates the effect of various materials, speeds, and pressures on the depth and volume of the wear scar.
- Range shows the numerical limits of each considered variable.
- Unit displays the unit of measurement for each variable.
- Type is one of three possibilities for the nature of the variable: Numerical, categorical, or ordinal [21]. The latter is not present in the experimental dataset. Speed and pressure are obviously numerical.

**Figure 2:**
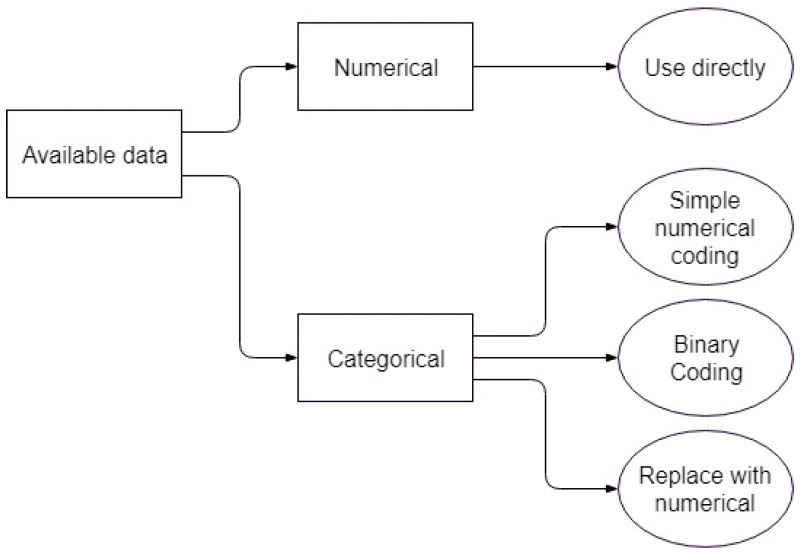
Flowchart for using available data.

For the material type, there are three possible options since a fitting application cannot work with simple labels as inputs. The first is not considered proper, and is only shown as a comparison to the other two. The second and third options lead to separate optimization problems, both of which are considered in parallel for each of the models involved. Figure 2 shows a graphical representation of the options available in the current context, while Table 2 briefly outlines the benefits and drawbacks of each option.

The first is coding the categorical variable as a simple numerical variable, with different integer numbers for each of the labels. For example, each of the material combinations used in the experiments can be assigned an integer, transforming it into a discrete numerical variable and allowing it to interact with the rest of the independent numerical variables.

AVE+30% SGF;C120 steel

AVE+30% SGF;Rp3 steel

Lexan+20% SGF;C120 steel

Noryl+20% SGF;C120 steel

However, this suffers from implicitly ordering the labels, which would negatively impact the model and possibly introduce hidden biases, since the assigned numbers have no mathematical meaning [21].

The second option is coding the categorical variable into multiple binary variables, one for each of the material labels. This is especially advantageous in the present context, since each value of the categorical variable is logically a combination of two materials, the scratched surface and the object used for scratching. As an aside, this was also possible for the first representation, but it further increased the danger of misrepresentation through the interaction of numerical labels. The second representation is shown in Table 3.

Each of the materials that make up the labels of the categorical variable will be binary variables themselves. The binary representation translates naturally to whether that particular material is present or not. This is the standard representation and is implemented in some of the models. The main drawback is that the scarcity of the resulting matrices may lead to rank deficiency in some of the methods for automatically generating fuzzy inference systems (genfis). Table 4 shows an excerpt of the available date for training, using this coding option.

The third option is replacing the categorical variable altogether. Instead of using the material labels, the model will introduce, as independent variables, the numerical characteristics of the various materials. To this end, five new variables for the polymers—specific weight, water absorption, elasticity, thermal conductivity and linear dilation—and three for the steel—S_max_, P_max_ and Ni_max_—are introduced. The chosen variables have a full complement of values for each of the materials used in the experiment. The eight new variables are shown in Table 5 and Table 6.

This gives the dataset eight independent variables that describe the variation in material on both sides of the experiment. The higher dimensionality is a slight disadvantage, but the representation is more natural due to them being actual numerical variables. An excerpt of the dataset is shown in Table 7 (numbers and labels are heavily truncated to allow representation).

In conclusion, the first coding option—simple numerical coding—is not suited to the task, while the second and third options are both used. This will create two distinct learning problems, which will be called the binary coding problem (option 2) and the numerical coding problem (option 3), on which the various algorithms are run. The results are shown and discussed for both cases in the appropriate sections.

**Figure 3:**
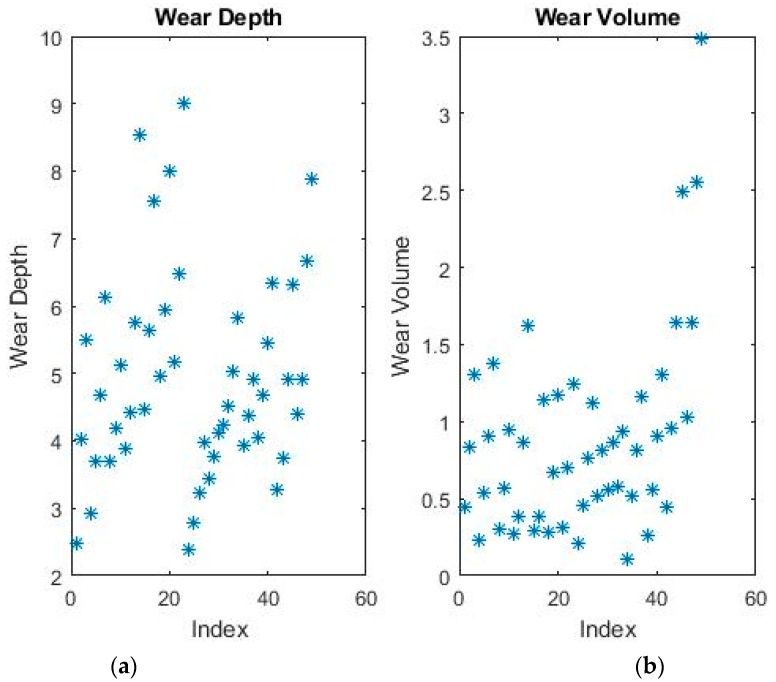
Dependent variables plotted on index for (**a**) wear depth and (**b**) wear volume.

An example of plotted data is shown in Figure 3. Due to the increased dimensionality, the data cannot be plotted as a graph dependent on the independent variables. Therefore, a two-dimensional graph based on the index was chosen instead. For the same reason, the data now has no discernible outliers, as can be seen in the figure, so all pre-processing conditions were eliminated.

The available data-points were randomly divided into a training set and a test set, with ratios of 70% of the total and 30% of the total, respectively. This is done by constructing a random index of 70% true values on a vector with the same length as the number of lines (i.e., samples) in the dataset. The training and test sets are then easily separated based on this index. The training test is used for optimizing the parameters of the various models including hyper-parameters (i.e., cross-validation). The test set is not available to any algorithm until the model is complete, when it is used to evaluate its performance on a heretofore unseen set of data belonging to the same phenomenon. Therefore, the evaluation of each model will take place both on the training set as well as on the test set.

## 3. Results

For metallic surface characterization on linear dry contact between the plastic material reinforced with SGF and alloyed steel, a multitude of experimental tests was performed with normal loads and different speeds, which led to the modelling of the metallic surface characterization on linear dry contact between the plastic material reinforced with SGF and alloyed steel using four predictor models presented below.

### 3.1. Linear Regression

The first model is a first-order multivariate linear regression optimized using batch gradient descent. The results obtained here will be used as a baseline for all other models. The optimization problem is described as $Y=\hat{\theta }*X$, where *X* is the input data, namely the dependent variables, containing all data-points, plus an intercept term. *Y* is the output data, alternatively the wear speed or wear volume, that the algorithm is trying to learn, and *θ* is the matrix of coefficients used to estimate *Y* from *X*. The challenge is finding the best coefficients, which minimizes the error between the actual *Y* and the estimate. This is obtained from $\hat{Y}=\theta_{LR}*X$, or, in extended form:

(1) $$\left[\begin{array}{c} \hat{y_{1}}\\ \hat{y_{2}}\\ \begin{array}{c} \hat{y_{3}}\\ \vdots \\ \hat{y_{n}} \end{array} \end{array}\right]=\left[\begin{array}{ccc} \theta_{11} & \cdots & \theta_{1\left(m+1\right)}\\ \vdots & \ddots & \vdots \\ \theta_{n1} & \cdots & \theta_{n\left(m+1\right)} \end{array}\right]\left[\begin{array}{c} x_{1}\\ x_{2}\\ \begin{array}{c} \vdots \\ x_{m}\\ int \end{array} \end{array}\right]$$

There will be *m* features and an intercept term, which helps prevent over-fitting. The total sum of all errors, across all values, is defined as the cost function. It is this cost function that the optimization algorithms attempt to minimize.

Gradient descent is an optimization algorithm where the potential solution is improved each iteration by moving along the feature gradient in the variable space. While it requires that the target function be differentiable and it is somewhat susceptible to local minima, gradient descent provides a stable and computationally inexpensive algorithm for function optimization.

As noted in the previous chapter, both representations of the dataset, using the two options for coding the categorical variable, were investigated in parallel. After running the gradient descent algorithm, the following coefficients were obtained, as shown in Table 8. Running the algorithm takes between 1 and 2 s for each of the dependent variables—the last run was timed internally at 1.174 s.

The obtained models will now predict the depth and volume of the wear scar as the linear combination of the vector of thetas and the value of a given feature set.

For example, using binary coding, the wear depth will be predicted as

(2)  D= 0.547+0.034Sp+0.096P+0.261A+0.335L−0.326N+0.018C−0.273R

**Figure 4:**
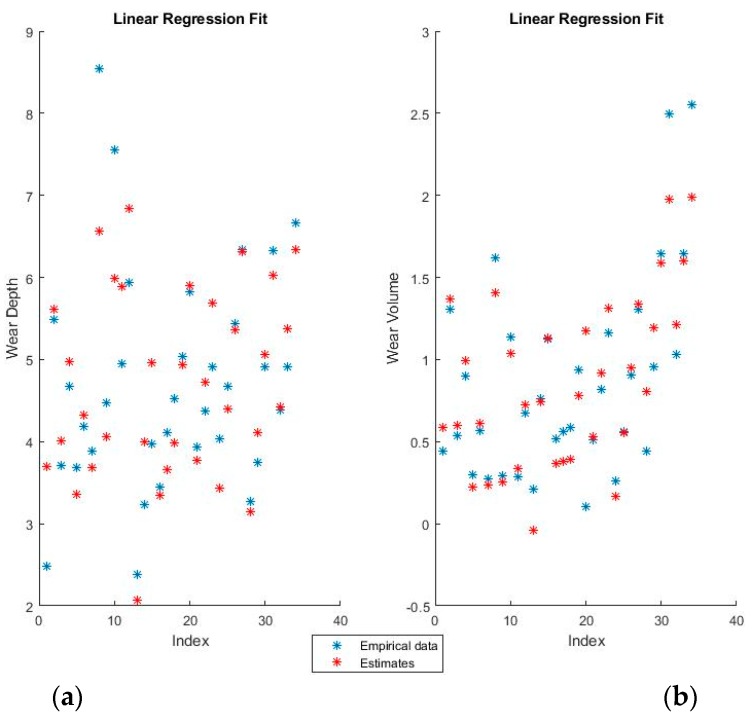
Linear regression fit for (**a**) wear depth and (**b**) wear volume in binary coding.

**Figure 5:**
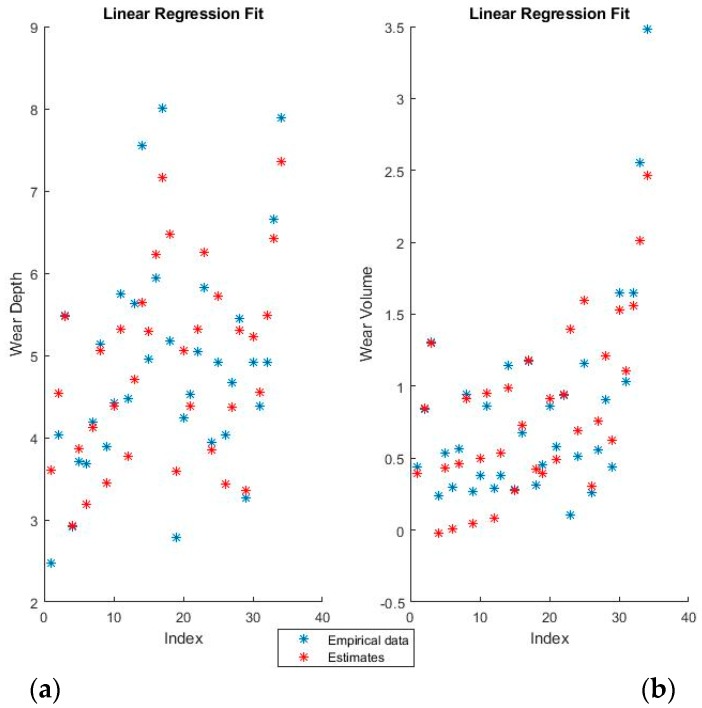
Linear regression fit for (**a**) wear depth and (**b**) wear volume in numerical coding.

Figure 4 and Figure 5 show the training fits for wear and volume, with both coding options, using linear regression.

The blue points represent the actual experimental data available for training, while the red points are the estimates that the model would obtain for the same input data.

The linear regression model has a decent performance of fitting the training data, but is obviously at a disadvantage because of the non-linearity present in the dataset. One interesting point of note is that the relative influence of the various features can be directly ascertained from the theta values, which shows both the magnitude and the direction of the dependency.

### 3.2. Neural Networks

**Figure 6:**
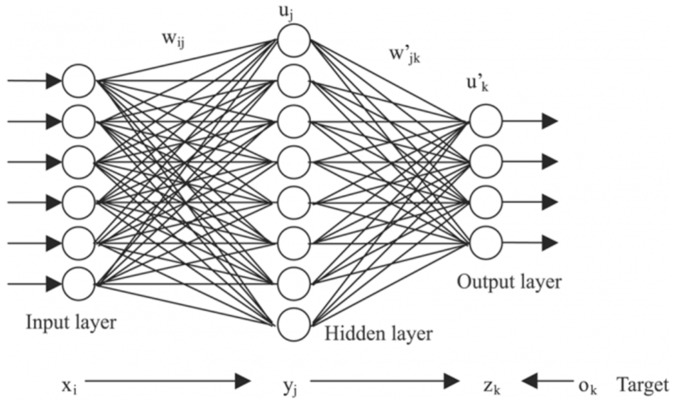
Artificial Neural Network with one hidden layer.

Neural network models are centered on a layer of hidden features (i.e., neurons) which control the prediction. Neural networks have an input layer that matches the considered independent variables and an output layer which matches the dependent variable. The model is optimized by successively tuning the weights associated to these neurons as well as their activation functions. A standard neural network is exemplified in Figure 6 [22].

The most important hyper-parameter of a neural network model is the number of neurons in the hidden layer. For the purposes of this application, all models contain 25 hidden neurons. This number was selected after running the algorithm with various levels of neurons and settling on the best performance in terms of the correlation coefficient for both the training and test sets. The training time varies greatly with the choice of training algorithm selected: Some may require only 1–2 s, while some configurations can take up to 30 s. The neural network selected here was timed at 12 s.

**Figure 7:**
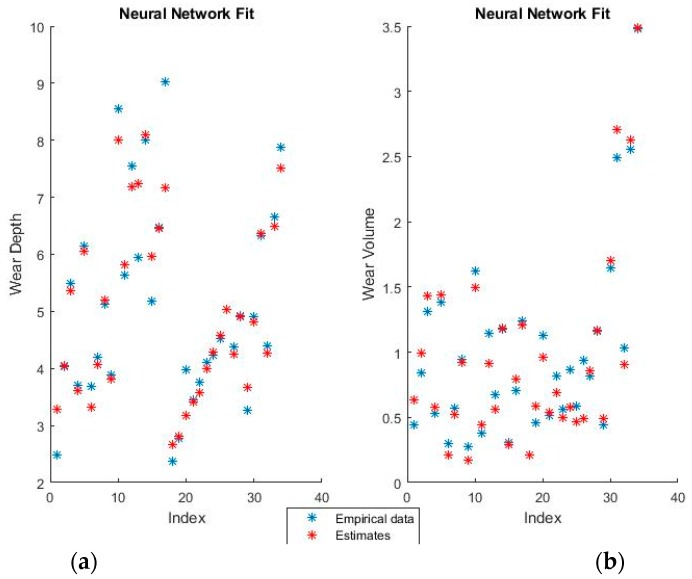
Neural Network fit for (**a**) wear depth and (**b**) wear volume in binary coding.

**Figure 8:**
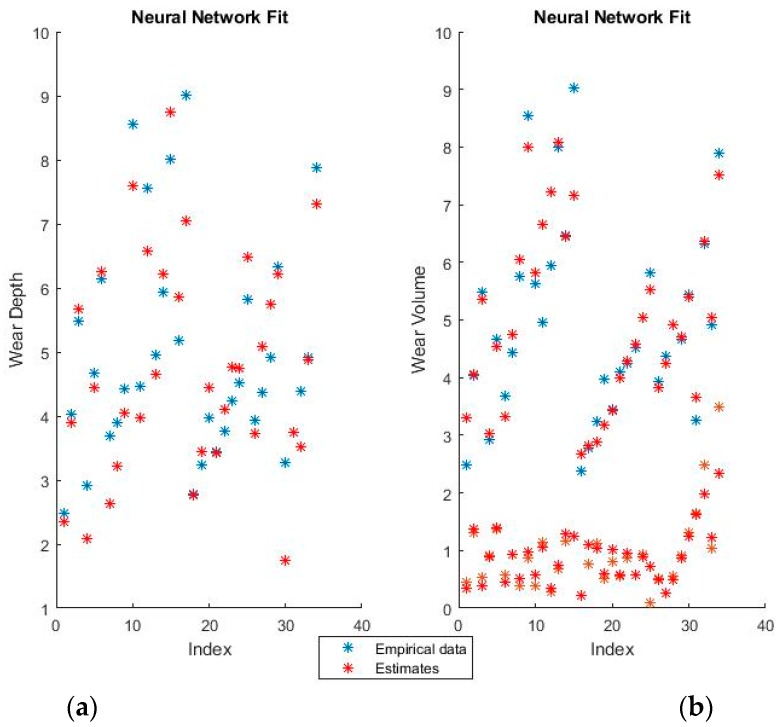
Neural network fit for (**a**) wear depth and (**b**) wear volume in numerical coding.

The weights of the selected neural networks are far too numerous to display in the paper. For example, to pass from the input layer of 10 neurons (10 features in the numerical coding case) to the hidden layer of 25 neurons, a matrix of 25 lines and 10 columns is required. Figure 7 and Figure 8 show the training fits for wear and volume with both coding options using a neural network model.

As with the linear regression figures, the blue points are the empirical data and the red points the estimates. It should be noted that perfectly correct estimates (red points) will overwrite the empirical data representation (blue points) where they coincide.

Neural networks give a very good fit of the training data. For example, notice how virtually all empirical points in the lower half of the volume fit, as seen in the graphical representation, have been overwritten by their estimates. Such good behavior on the training set, however, always raises suspicions of over-fitting, which will be verified or invalidated when the model is used on the training set.

### 3.3. Generated Fuzzy Inference System

Fuzzy inference systems can be automatically generated and then used as actual models for predicting the future behavior of a system. The generated FIS can then be deployed either as such, or further optimized using an ANFIS algorithm, which will be discussed in the next section. As these tend to be computationally intensive models, the FIS is of the Sugeno type, which employs linear or constant functions as outputs, as opposed to the Mamdani type FIS, whose outputs are also fuzzy membership functions. This type of FIS has fewer parameters and is therefore somewhat less computationally intensive. As will be discussed in the final results section, there is also the danger of over-fitting since there are so many parameters and comparatively few data-points.

Given a set of raw data, there are three options for obtaining a working FIS: Grid partitioning (genfis1), subtractive clustering (genfis2), and fuzzy c-means clustering (genfis3). There are a number of hyper-parameters to be tuned such as the number of membership functions per input and the shape of each membership functions. These were chosen after some trials to be four membership functions per numerical variable and two per binary variable, when dealing with the binary coding option. The shape of the membership functions was kept as standard bell curves (Gaussian distributions). Generating fuzzy inference systems is performed quickly, usually within a second. The last run yielding internal timings of 0.177 s for genfis1, 0.153 s for genfis2, and 0.248 s for genfis3.

**Figure 9:**
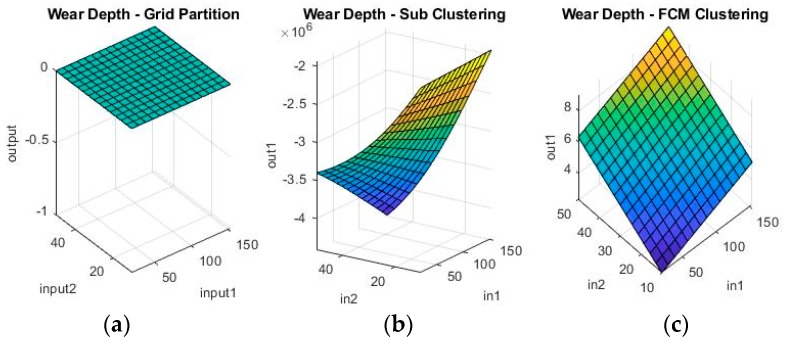
Fuzzy inference systems generated through (**a**) grid partitioning; (**b**) sub-clustering; and (**c**) Fuzzy c-Means (FCM) clustering for wear depth in numerical coding.

**Figure 10:**
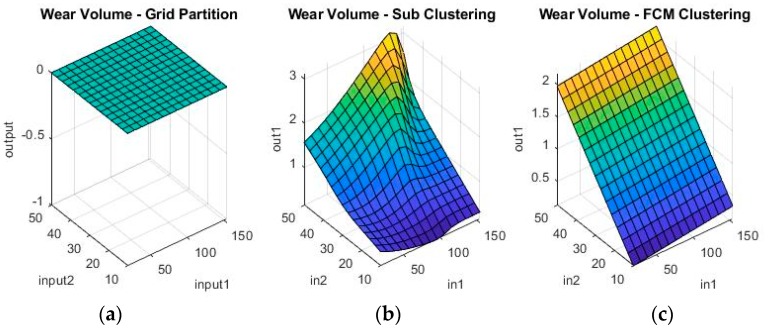
Fuzzy inference systems generated through (**a**) grid partitioning; (**b**) sub-clustering; and (**c**) FCM clustering for wear volume in numerical coding.

Grid partitioning is really only used as a benchmark, since it is commonly held that its performance is unsatisfactory unless further developed with ANFIS [23,24,25]. However, because it is such a rudimentary starting point for the ANFIS algorithm, it will actually not work on the numerical coding options, as there are simply too few data-points, too many parameters and too computationally intensive a task. Therefore, ANFIS starting from grid partitioning is only investigated for the binary coding problem. The other two options are, however, valid possible solutions both on their own, as well as after further optimization. Figure 9 and Figure 10 show a sample of the fuzzy inference spaces of the FISs obtained through automatic generation. Since it is impossible to show the n-dimensional graph of the FIS, the first two inputs are chosen as the independent variable axis.

**Figure 11:**
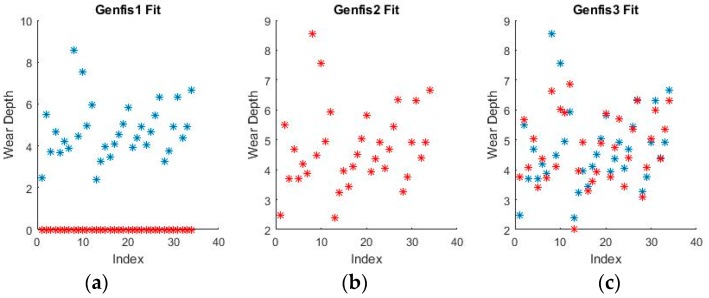
Fuzzy interference systems (FIS) fit generated through (**a**) grid partitioning; (**b**) sub-clustering; and (**c**) FCM clustering for wear depth in binary coding.

**Figure 12:**
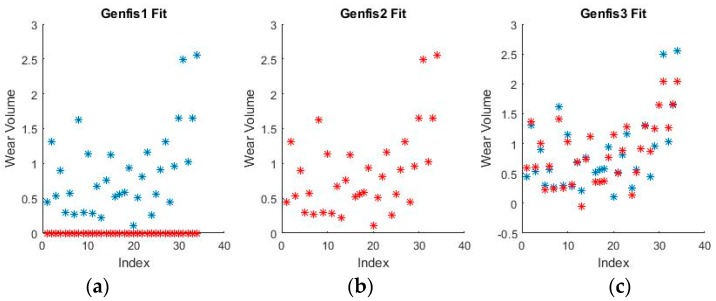
FIS fit generated through (**a**) grid partitioning; (**b**) sub-clustering; and (**c**) FCM clustering for wear volume in numerical coding.

As can be seen from Figure 9 and Figure 10, grid partitioning provides a simple starting point for further optimization, while sub-clustering and FCM clustering present very interesting fuzzy inference spaces. Figure 11 and Figure 12 show a selection of the fit obtained using the generated fuzzy inference systems.

From the spread of the empirical and estimated data, some early conclusions can be drawn about generated fuzzy inference systems. As already discussed, grid partitioning is simply an empty form for a FIS, which is the starting point of an ANFIS algorithm, as no attempt is made to fit the experimental data. The FIS obtained through subtractive clustering has near perfect performance on the training set, which naturally raises concerns about the possibility of over-fitting the data. These will be addressed when performing on the test set. The third method, fuzzy c-means clustering, obtains good, if not great, performance and is a very good start for further optimization.

### 3.4. Adaptive Neuro-Fuzzy Inference Systems

ANFIS uses back propagation to determine the premise parameters of each rule. The consequent parameters are then determined using a least mean squares algorithm. An iteration of the learning algorithm consists of two passes: In the forward pass, the premise parameters are fixed and the consequent parameters are optimized through an iterative least squares approach, while going through the rule base system. In the backward pass, the consequent parameters are fixed, while the premise parameters are modified using back propagation. This algorithm continues until the target error is met or the number of iterations exceeds a predetermined threshold. An excellent description of the ANFIS architecture and learning procedure is given by Denai et al. [26].

Adaptive neuro-fuzzy inference systems construct a FIS capable of predicting the values of the dependent variables through further optimization of a generated FIS structure. If no initial FIS is specified, one is created through a genfis1 (grid partitioning) algorithm. With the exception of grid partitioning in the case of numerical coding, as discussed previously, all generated FISs were run through an ANFIS algorithm in the hopes of improving performance. The algorithm is relatively fast, usually lasting a few seconds. It will take longer when starting from a FIS obtained through grid partitioning (genfis1), since it requires more optimization. The inference systems discussed in the paper were timed at 4.987 s for ANFIS 1, 1.019 s for ANFIS 2, and 1.866 s for ANFIS 3.

**Figure 13:**
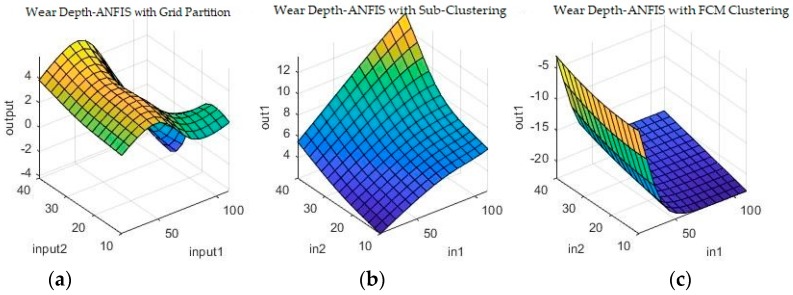
Fuzzy inference systems generated through adaptive neuro-fuzzy inference systems (ANFIS) with (**a**) grid partitioning; (**b**) sub-clustering; and (**c**) FCM clustering for wear depth in binary coding.

**Figure 14:**
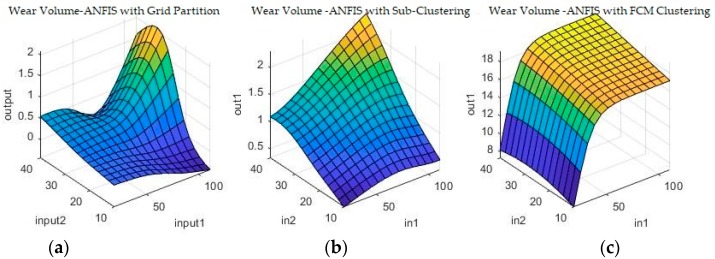
Fuzzy inference systems generated through ANFIS with (**a**) grid partitioning; (**b**) sub-clustering; and (**c**) FCM clustering for wear volume in binary coding.

The algorithm was used with a standard set of hyper-parameters such as 50 generations and the inherited FISs obtained at the previous step. Figure 13 and Figure 14 show a selection of the resulting fuzzy inference spaces.

**Figure 15:**
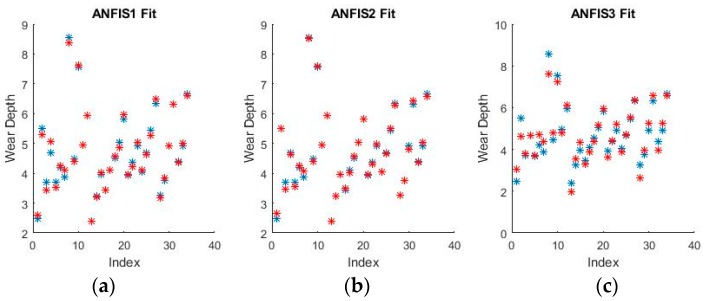
ANFIS fit generated through (**a**) grid partitioning; (**b**) sub-clustering; and (**c**) FCM clustering for wear depth in binary coding.

**Figure 16:**
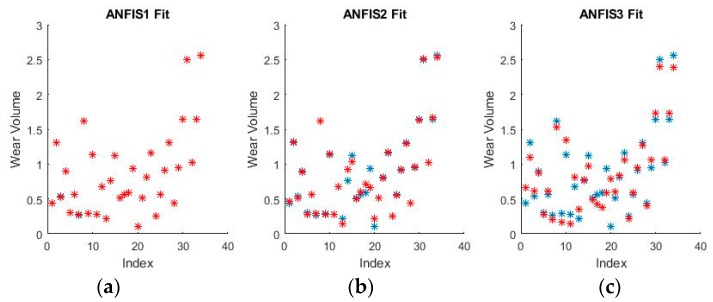
ANFIS fit generated through (**a**) grid partitioning; (**b**) sub-clustering; and (**c**) FCM clustering for wear volume in binary coding.

The fuzzy inference spaces obtained after running the algorithm have a very interesting configuration. The resulting rule base spaces are clearly nonlinear, which should lead to a good ability to fit the present dataset. Figure 15 and Figure 16 show a selection of the resulting performance when fitting the training set data. 

The performance shown by all ANFISs is very encouraging as the spreads shown above seem to reveal very good behavior from all systems. Pending the requisite investigation on the test sets, the ANFISs appear to be the frontrunners for fitting the experimental dataset. Of particular note is the near perfect performance obtained in fitting the wear volume.

### 3.5. Model Results

While the figures provide a good overview of the models’ behavior, they fail to give an analytical measure of model performance. For this, two metrics were used, both of which gave a numerical measure of the error: The mean square error (MSE) and the mean absolute error (MAE). Since these are metrics for the error of the model, lower values correlate to better performance. The mathematical expressions are shown below:

(3) $$\mathrm{MSE}=\frac{1}{n}\underset{i=\bar{1\ldots n}}{\sum }{\left(X_{i}*\theta -Y_{i}\right)}^{2}$$

(4) $$\mathrm{MAE}=\frac{1}{n}\underset{i=\bar{1\ldots n}}{\sum }\left|X_{i}*\theta -Y_{i}\right|$$

These two error functions should provide the definitive information on which model best fits the available experimental data. The results are put together into Table 9 and Table 10 below, one for each dependent variable.

It should first be noted that there is a random element to the above results. No two algorithm runs result in exactly the same models, hence there is some variation in the implementation of the simulation. However, the algorithms have been run multiple times and each set of results supports the general conclusions which are to be drawn from the above tables.

The obtained prediction function provides a good fit of the model, with little error and no over-fitting, as can be concluded from the testing phase of the algorithm. Once trained, the predictive model can be used instead of the actual analytical approach in any application where the dependent variables are needed and the independent variable values are available, within a similar context. This reduces the mathematical complexity of the overall application and could find use in a range of computationally intensive models. It is these functions that will be further improved through the addition of more data as it becomes available, and will be used to predict the future behavior of the experiment.

## 4. Discussion

The discussion of results revolves around the test scores for the two types of error functions obtained by each model.

In judging the various algorithms, a lower value for MSE and MAE norms are preferable since these represent errors in fitting. For example, in modelling the wear volume with binary coding, the ANFIS2 model is preferable to the linear regression as it achieves lower error grades in both MSE and MSE for the training suite (0.004 < 0.067 and 0.033 < 0.159) as well as the test suite (0.02 < 0.095 and 0.11 < 0.17). Furthermore, it is relatively easy to spot an over-fitting model as it will have good to great performance on the training suite, but poor performance on the test suite. This means it fits the available training data too closely and is ill-equipped to handle new incoming data from the same experiment. As an example, Genfis2 fits the training data for wear volume in binary coding perfectly—the errors are actually zero, but it has very large errors when used on the test data (MSE = 1453 and MAE = 10.48).

As relates to the coding option being used, the variation in results from binary to numerical coding has generally been minimal. While the numbers are obviously different, the same tendencies exist in both representations of the problem.

Linear regression actually performs very well on the test set and in fact overall, suggesting there may be some linear dependencies between the dependent and independent variables after all. It does not provide the level of performance of the neural network or ANFIS models, but as a baseline model, it does very well.

The neural network models have the best overall performance in terms of being the most balanced. While occasionally overtaken by some of the ANFIS or even Genfis algorithms, the neural network models always give very good performance, significantly overtaking the baseline models (i.e., the linear regression models).

The Genfis models achieved mixed results. While sometimes showing superb performance, overtaking even the neural networks or some ANFIS models, they are susceptible to dangerous levels of over-fitting. When that happens, the model is virtually useless as it achieves disproportionate error levels on the test sets—see Genfis2 for wear volume fitting in binary coding. Genfis1 was never meant to be an actual fitting model and of course performs the most poorly, although it does have a few instances when it is workable. However, these should be seen as chance encounters with a low-value dataset, rather than indicative of possible improved performance. As has been mentioned, Genfis1 is only meant to provide a starting point for further optimization. Genfis2 and 3 perform very well, sometimes challenging the performance of NN or ANFIS algorithms, with the aforementioned caveat of possibly disastrous over-fitting. It should also be noted that, in the binary case, Genfis is greatly helped by being able to declare two, membership, function variables, which very nicely model the binary ones.

ANFIS models performed very well throughout all tests and are the main contender of the neural network algorithms, which they oftentimes outperformed. The variation of their results was slightly higher than that of the neural networks, which is their only real disadvantage. However, given the inherent variation of results from different runs of the algorithms, it can be said that their performance is comparable and, in some instances, preferable, to that of the neural networks. Additionally, similar to neural networks and in contrast to some of the Genfis models, they do not seem to be significantly affected by over-fitting, even though they are constructed on rather complex structures.

The elapsed times of the various algorithms are generally comparable, lasting in the range of a few seconds, with the exception of training neural networks under certain configurations. While less training time is always preferable, it is not a major factor for the selection of this model, since the envisioned applications do not require real-time performance. As more experimental data becomes available, the elapsed times will likely increase and future work will deal with improving the speed of all the algorithms discussed in the paper. As a final recommendation and conclusion of the results presented, both neural networks and ANFIS (type 2 and 3) models perform very well on both the training and test sets and could be used for implementation. Any further choice between these two architectures would have more to do with either personal preference or external requirements for an eventual expanded application.

## 5. Conclusions

The studies on modelling the friction and wear of metallic surfaces for friction couples with linear contact between a thermoplastic material armed with short glass fibers (SGF) and steel are complex and outline the influence of input parameters specific to the tribological system (normal load: contact pressure, the relative sliding speed, the friction type, and the characteristics of the materials in contact) on the output parameters of the tribological system: The wear volume of metallic material and the depth of the wear material.

Neuro-fuzzy modelling of the metallic surface characterization on linear dry contact between plastic material reinforced with SGF and alloyed steel proves that the friction force is not only dependent on the friction coefficient and normal load, as previously thought, but also on the sliding speed and the physical and mechanical properties of the materials, which has significant impact in the characterization of new industrial processes utilizing short glass fiber reinforced polymers.

Using advanced statistical and optimization algorithms on a dataset obtained from the hardware simulation, the results of the research led to modelling a dependency between the various variables of interest involved in the friction process. The subject draws a growing interest from the research community with the advent of highly advanced, intelligent classification, and optimization and regression algorithms.

This research focused on processing an experimental dataset on contact wear, with the aim of obtaining prediction models for the two variables of interest, wear depth, and wear volume. The data was obtained through experiments run on friction couples with linear contact using three different types of polymers on two different types of steel variants. Aside from alternating the materials used, the speed and pressure applied to them were varied under the same operating conditions. The data was pre-processed and coded for use with numerical learning algorithms. Since two viable coding options were found, both resulting numerical problems were treated separately. Various possible data fitting models were then investigated, including a baseline model for reference (linear regression) and different variation of possible considered structures (Genfis and ANFIS models). These models were then judged on their ability to fit the available data, as quantified by two standard error norms, calculated for each model. The end result is a comparison of the performance of the multiple prediction systems and a discussion of the various factors that influence these numerical results.

The chosen models are to be implemented in future work and in any practical implementation in similar conditions for metallic surface characterization within the field of tribology, robotics, and with wide impact metallic surface characterization applications in the wear process, emphasizing the abrasive, adhesive, and corrosive wear. It will be of great interest to investigate the interaction of the current prediction models with real world applications for which there are many options [6,11,12,27].

Another very important direction for further research focuses on gathering more experimental data to allow the development of even more complex enhanced models, either pertaining to new developments in the state of the art, or for retraining the current architectures. Over-fitting, an issue present and discussed throughout the work, should be greatly diminished with training on more available data. This is because most of the models do not over-fit out of design or implementation reasons, but simply because some are too complex (i.e., including too many parameters) for the current dataset.

## Figures and Tables

**Table 1:** Dataset.

Variable	Mode	Type	Range	Unit
Material	Independent	Categorical	N/A	N/A
		Numerical	-	-
Speed	Independent	Numerical	18.56–153.55	cm/s
Pressure	Independent	Numerical	10–50	N
Depth	Dependent	Numerical	0.9–9.1	10^−4^ mm^3^
Volume	Dependent	Numerical	0.13–3.48	10^−4^ mm^3^

**Table 2:** Comparison of coding options.

Option No.	Coding	Benefits	Disadvantages
1	Simple Numerical Coding	Simplest implementation	Implicitly orders labels, introducing bias.
2	Binary Coding	Easy to implement. Translates naturally to actual context.	Introduces multiple new variables. May cause rank deficiencies when working with matrices.
3	Replace with numerical	Leads to a completely numerical problem. Eliminates the issues in simple and binary coding.	Introduces many new variables. May cause dimensionality problems with small datasets.

**Table 3:** Binary coding of variables.

Material	AVE	Lexan	Noryl	C120	Rp3
AVE + 30% SGF with C120 steel	1	0	0	1	0
AVE + 30% SGF with Rp3 steel	1	0	0	0	1
Lexan + 20% SGF with C120 steel	0	1	0	1	0
Noryl + 20% SGF with C120 steel	0	0	1	1	0

**Table 4:** Dataset excerpt using binary coding.

Independent								Dependent	
Material	Speed	Pressure	A	C	R	L	N	Depth	Volume
AVE + 30% SGF/C120	18.56	20	1	1	0	0	0	2.4798	0.4404
AVE + 30% SGF/C120	27.85	20	1	1	0	0	0	3.7076	0.5338
AVE + 30% SGF/C120	37.13	30	1	1	0	0	0	5.1336	0.9418
AVE + 30% SGF/C120	46.41	10	1	1	0	0	0	3.8871	0.2714
AVE + 30% SGF/C120	111.4	10	1	1	0	0	0	4.9482	0.283
AVE + 30% SGF/Rp3	18.56	40	1	0	1	0	0	3.9708	1.1247
AVE + 30% SGF/Rp3	27.85	20	1	0	1	0	0	3.4464	0.5164
AVE + 30% SGF/Rp3	37.13	30	1	0	1	0	0	4.2392	0.8627
AVE + 30% SGF/Rp3	46.41	20	1	0	1	0	0	4.5242	0.5833
AVE + 30% SGF/Rp3	46.41	30	1	0	1	0	0	5.0392	0.9377
Lexan + 20% SGF/C120	27.85	40	0	1	0	1	0	4.9169	1.1594
Lexan + 20% SGF/C120	46.41	10	0	1	0	1	0	4.0361	0.2582
Noryl + 20% SGF/C120	46.41	40	0	1	0	0	1	6.3271	2.4946
Noryl + 20% SGF/C120	55.7	20	0	1	0	0	1	4.3885	1.0289
Noryl + 20% SGF/C120	55.7	30	0	1	0	0	1	4.9133	1.6474

**Table 5:** Polyamide characteristics.

Polyamide	Weight	Absorption	Elasticity	Conductivity	Dilation
AVE	1.35	0.8	80	0.34	3.3
Noryl	1.27	0.06	84	0.196	2.5
Lexan	1.35	0.16	86	0.5	2.68

**Table 6:** Steel characteristics.

Steel	S_max_	P_max_	Ni_max_
Rp3	0.02	0.025	0.4
C120	0.025	0.03	0.35

**Table 7:** Dataset excerpt using numerical coding.

Independent											Dependent	
Material	Sp	Pr	Wgt	Abs	Els	Cd	Dil	S	P	Ni	Dpt	Vol
A/C120	18.5	40	1.35	0.8	80	0.34	3.3	0.025	0.03	0.35	5.48	1.30
A/C120	27.8	10	1.35	0.8	80	0.34	3.3	0.025	0.03	0.35	2.91	0.23
A/C120	27.8	40	1.35	0.8	80	0.34	3.3	0.025	0.03	0.35	6.13	1.37
A/C120	37.1	10	1.35	0.8	80	0.34	3.3	0.025	0.03	0.35	3.68	0.29
A/C120	46.4	40	1.35	0.8	80	0.34	3.3	0.025	0.03	0.35	8.54	1.62
A/C120	57.7	10	1.35	0.8	80	0.34	3.3	0.025	0.03	0.35	4.47	0.29
A/C120	111	30	1.35	0.8	80	0.34	3.3	0.025	0.03	0.35	8.00	1.17
A/C120	153	10	1.35	0.8	80	0.34	3.3	0.025	0.03	0.35	5.17	0.31
A/Rp3	18.5	40	1.35	0.8	80	0.34	3.3	0.02	0.025	0.4	3.97	1.12
A/Rp3	27.8	20	1.35	0.8	80	0.34	3.3	0.02	0.025	0.4	3.44	0.51
A/Rp3	37.1	30	1.35	0.8	80	0.34	3.3	0.02	0.025	0.4	4.23	0.86
A/Rp3	46.4	20	1.35	0.8	80	0.34	3.3	0.02	0.025	0.4	4.52	0.58
L/C120	27.8	40	1.35	0.16	86	0.5	2.6	0.025	0.03	0.35	4.91	1.15
L/C120	46.4	10	1.35	0.16	86	0.5	2.6	0.025	0.03	0.35	4.03	0.25
N/C120	46.4	40	1.27	0.06	84	0.19	2.5	0.025	0.03	0.35	6.32	2.49
N/C120	55.7	20	1.27	0.06	84	0.19	2.5	0.025	0.03	0.35	4.38	1.02

**Table 8:** Coefficient values from the linear regression algorithm.

Binary Coding			Numerical Coding		
Features	Thetas		Features	Thetas	
	Depth	Volume		Depth	Volume
Intercept	0.5470	−0.1621	Intercept	0.1632	4.5657
Speed	0.0339	0.0015	Speed	0.0313	0.0035
Pressure	0.0961	0.0391	Pressure	0.1095	0.0450
AVE	0.2615	−0.1706	Weight	0.0252	−0.1091
Lexan	0.3347	0.1097	Absorption	0.0826	−0.3794
Noryl	−0.3257	−0.1250	Elasticity	−0.0004	−0.0373
C120	0.0184	−0.2411	Conductivity	0.0394	−0.4060
Rp3	−0.2729	0.3989	Dilation	0.1059	−0.4695
			Smax	0.0197	0.0072
			Pmax	0.0188	0.0062
			Nimax	−0.1858	−0.0552

**Table 9:** Wear depth fitting metrics.

Model	Binary Coding				Numerical Coding			
	Train Suite		Test Suite		Train Suite		Test Suite	
	MSE	MAE	MSE	MAE	MSE	MAE	MSE	MAE
Linear	0.38528	0.46629	0.64289	0.54639	0.54483	0.53454	0.49382	0.50348
Neural	0.07182	0.18512	0.29991	0.30002	0.52048	0.55583	0.19614	0.33355
Genfis1	26.306	4.92209	27.094	4.93573	29.5935	5.19193	19.64452	4.32411
Genfis2	0.01578	0.07577	0.19928	0.33022	0.02053	0.09137	0.27421	0.41283
Genfis3	0.38405	0.47340	0.56615	0.59409	0.40373	0.47233	0.51991	0.59842
ANFIS1	0.02162	0.10473	2.98506	0.78510				
ANFIS2	0.02265	0.11829	0.30524	0.43454	0.04433	0.15980	0.16904	0.35767
ANFIS3	0.19765	0.34180	0.42079	0.49680	0.11747	0.29467	0.50163	0.62247

**Table 10:** Wear volume fitting metrics.

Model	Binary Coding				Numerical Coding			
	Train Suite		Test Suite		Train Suite		Test Suite	
	MSE	MAE	MSE	MAE	MSE	MAE	MSE	MAE
Linear	0.06750	0.15916	0.09580	0.17017	0.11225	0.20713	0.08110	0.19386
Neural	0.02062	0.10657	0.01620	0.10758	0.08371	0.17435	0.04756	0.14160
Genfis1	1.09606	0.86541	1.45574	0.93736	1.49774	0.98549	1.37690	0.91272
Genfis2	0.00000	0.00000	1435	10.48023	0.00965	0.03834	1.82954	0.56139
Genfis3	0.06648	0.15842	0.03457	0.11739	0.07857	0.19069	0.04393	0.16326
ANFIS1	0.00000	0.00017	1.63150	0.47196				
ANFIS2	0.00441	0.03373	0.02079	0.11009	0.00965	0.03821	0.00435	0.04772
ANFIS3	0.02919	0.11852	0.05496	0.20210	0.00002	0.00182	0.00043	0.01699
